# Immunopathogenesis of HPV-Associated Cancers and Prospects for Immunotherapy

**DOI:** 10.3390/v9090254

**Published:** 2017-09-12

**Authors:** Sigrun Smola

**Affiliations:** Institute of Virology, Saarland University Medical Center, 66421 Homburg/Saar, Germany; sigrun.smola@uks.eu; Tel.: +49-6841-16-23931

**Keywords:** human papillomavirus, cervical cancer, skin cancer, epidermodysplasia verruciformis, immune evasion, chronic inflammation, IL-6, JAK-STAT3, immunotherapy, immunoscore

## Abstract

Human papillomavirus (HPV) infection is a causative factor for various cancers of the anogenital region and oropharynx, and is supposed to play an important cofactor role for skin carcinogenesis. Evasion from immunosurveillance favors viral persistence. However, there is evidence that the mere presence of oncogenic HPV is not sufficient for malignant progression and that additional tumor-promoting steps are required. Recent studies have demonstrated that HPV-transformed cells actively promote chronic stromal inflammation and conspire with cells in the local microenvironment to promote carcinogenesis. This review highlights the complex interplay between HPV-infected cells and the local immune microenvironment during oncogenic HPV infection, persistence, and malignant progression, and discusses new prospects for diagnosis and immunotherapy of HPV-associated cancers.

## 1. Introduction

Approximately 15–20% of all cancers are caused by infectious agents [1] and around 5% by human papillomaviruses (HPVs) [2,3]. The causal relationship between HPV infection and cervical cancer, which harbors HPV in up to 99.7% of cases [4], was highlighted by Harald zur Hausen, who was awarded a Nobel Prize in 2008. In addition to cervical cancer, a significant number of oropharyngeal, penile, anal, vaginal and vulvar cancers are induced by mucosal HPVs [5,6,7] and cutaneous HPVs have been implicated as cofactors in skin cancer development [8,9].

Invasive cancer is not an immediate consequence of HPV infection. HPV-induced carcinogenesis takes years or decades to occur, and there is increasing evidence that additional tumor-promoting steps are required [10]. It is widely accepted that effective immune control is required to prevent persistent HPV infection. Recent studies indicate, however, that chronic inflammation and misled immune responses in the local immune microenvironment play a critical role during the progression of precancerous lesions to invasive cancer [11,12,13]. Thus, the unidirectional view of an immune system that primarily serves to attack and eliminate HPV-infected and neoplastic cells needs to be revised [14].

While screening programs have greatly reduced the burden of cervical cancer in developed countries, current diagnostic tests cannot discriminate between lesions that will progress to invasiveness and those that do not. This results in an overtreatment of high-grade lesions that are detected during screening [15]. A better understanding of the immunological mechanisms contributing to HPV-associated cancer development would likely propel not only more accurate diagnosis of progressing precancerous lesions but also novel immunotherapeutic approaches for HPV-driven cancers [16].

This review focuses on the current understanding of the complex interplay between HPV-infected cells and the local immune microenvironment during HPV infection and HPV-associated carcinogenesis, and discusses novel prospects for diagnosis and immunotherapy.

## 2. Human Papillomaviruses in Mucosal versus Skin Carcinogenesis and Immune Control

Human papillomaviruses are non-enveloped double-stranded (ds) DNA-viruses that are transmitted sexually or by smear infection [17]. More than 200 different HPV types that are contained within 5 different genera have been characterized [18,19]. HPV-induced pathologies vary from benign warts and low- and high-grade neoplasia to malignant cancer and depend on respective HPV types as well as anatomical sites of infection [20]. While almost all cervical cancers are HPV-associated, 64–91% of vaginal, 40–50% of vulvar, 88–94% of anal and 40–50% of penile cancers are HPV-positive [3]. Notably, prevalences of HPV-driven oropharyngeal cancers display larger geographical variations. The highest prevalence is observed in developed countries, with HPV-positivity rates ranging from 35% up to more than 70% in some regions, and numbers of oropharyngeal cancers have significantly increased during the last decades [3,5]. 12–15 mucosal HPV types, all belonging to genus α, have been identified as so-called high-risk HPV (HR-HPV) types [21]. Numerous studies on the HPV life cycle and on the biology of the mucosal HPV oncogenes E6 and E7 have greatly improved our understanding of HPV-induced transformation far beyond the mere inactivation of the tumor suppressor proteins p53 and retinoblastoma (for a review see [22]).

Genus β-HPVs have been implicated in ultraviolet (UV) light-induced non-melanoma skin cancer in patients suffering from the inherited disease Epidermodysplasia verruciformis [23] and a cofactor role for skin carcinogenesis in the normal population has been discussed (for reviews see [8,9,24]). Recent data point to an early role of genus-β HPV in skin carcinogenesis. It has been shown that β-HPV type 8 infection expands the stem cell compartment in Epidermodysplasia verruciformis patients by suppressing the stemness-repressing microRNA-203 [25], an initial key step in skin carcinogenesis. Mechanistically, the E6 protein, the major oncoprotein of HPV8 [26], targets the transcription factor CCAAT/enhancer binding protein (C/EBP)α that serves as a tumor suppressor of UV-induced carcinogenesis. HPV8 E6 thereby prevents microRNA-203 expression, leading to potent up-regulation of the epithelial stemness-maintenance factor ∆Np63 (NH2-terminally deleted p63). ∆Np63 in turn promotes proliferation and inhibits differentiation of keratinocytes [25]. This is in contrast to the E6 protein encoded by mucosal HR-HPV, which suppresses miR-203 via interference with p53 [27], while the E7 protein suppresses a protein kinase C-dependent pathway [28]. Proliferation of hair follicle stem cells was also observed in transgenic mice expressing the HPV8 early region under the control of the keratin 14 (K14)-promoter [29]. Moreover, genus β-HPVs were shown to suppress UV-induced DNA damage repair; they interfere with Notch-signaling that further contributes to ∆Np63 up-regulation and have anti-apoptotic properties in vitro. Different β-HPVs have oncogenic potential in transgenic mice, particularly in synergism with UV-light exposure [26,30,31,32,33,34,35].

In mucosal as well as cutaneous HPV-associated carcinogenesis it is well accepted that the immune system has an important surveillance function. In immunocompetent individuals, up to 90% of anogenital HPV infections are cleared within two years [36,37,38] and this is thought to be due to innate immunity as well as adaptive CD8^+^ T cell-mediated responses directed against viral early proteins [39,40]. Conversely, patients with impaired adaptive immunity, such as transplant recipients or HIV-patients, show higher prevalences of HPV infection and HPV-related diseases, further underlining the importance of immunosurveillance in HPV-associated carcinogenesis [8,41,42,43,44,45,46].

**Figure 1:**
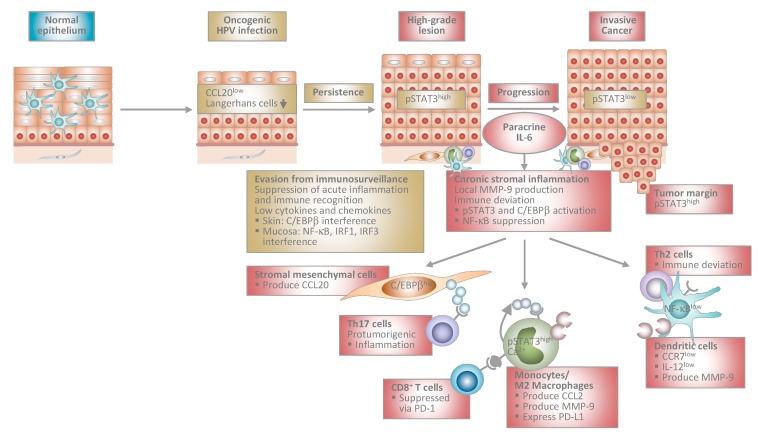
Proposed model of human papillomavirus-induced carcinogenesis. Stage-specific interplay between virally infected keratinocytes and the local immune microenvironment. At early stages, HPV-infected cells suppress acute inflammation in the epithelium and immune recognition. This allows escape from immunosurveillance and viral persistence. During progression to invasive cancer HPV-transformed cells initiate chronic stromal inflammation and immune deviation orchestrated by paracrine IL-6. The IL-6/STAT3 and IL-6/C/EBPβ pathways lead to chemokine induction in stromal mesenchymal and infiltrating immune cells. As a consequence, myelomonocytic cells expressing protumorigenic MMP-9 and Th17 cells are recruited further promoting inflammation. Myelomonocytic cells differentiate into functionally impaired dendritic cells or M2 macrophages expressing PD-L1 that inhibit cytotoxic T cell responses. IL-6 suppresses NF-κB activity in stromal dendritic cells, which are unable to migrate in response to lymph node homing chemokines due to low CCR7 chemokine receptor expression. Instead, they are immobilized within the tumor stroma and produce MMP-9 locally. IL-12 is expressed only at low levels shifting T helper cell responses from Th1 to Th2. Stromal inflammation and immune deviation facilitate progression to invasiveness. HPV: human papillomavirus; IL: interleukin; STAT3: signal transducer and activator of transcription 3; C/EBP: CCAAT/enhancer binding protein; MMP: matrix-metalloproteinase; Th: T helper; PD-L1: programmed death-ligand 1; NF: nuclear factor; CCR: C-C chemokine receptor; CCL: C-C chemokine ligand; IRF: interferon regulatory factor.

HPV infections that escape immune control can persist and a certain proportion progresses to cancer. Recent studies have shed light on the immune system as a double-edged sword in HPV-associated carcinogenesis and evidence is increasing that the role of the immune system changes in a stage-dependent manner. At earlier stages, anti-viral immunity predominates and the virus has adopted strategies to counteract immunosurveillance in order to establish persistence in the epithelium. However, at later stages of the disease, HPV-transformed cells reprogram the local immune microenvironment and rather initiate chronic stromal inflammation, which then serves to promote progression of precursor lesions to invasive cancer (Figure 1).

## 3. Immune Escape Paves the Way for HPV Persistence

To maintain a first line of defense against infections agents, skin and mucosal surfaces are equipped with efficient immune sentinels and immune effector mechanisms [47,48]. Keratinocytes, the HPV host cells, form stratified epithelia constituting a physical and immunological barrier against pathogens. They are armed with pathogen recognition receptors, host intrinsic restriction factors and an arsenal of inflammatory cytokines and chemokines orchestrating local immune responses [49]. While the epidermal compartment harbors Langerhans cells and distinct subsets of antigen-presenting cells (APCs) [50], most innate immune cell types including myeloid, dendritic as well as innate lymphoid cells and adaptive resident lymphocytes are located in the dermis [51].

### 3.1. Passive Mechanisms of Immune Escape

For productive infection HPV depends on the keratinocyte differentiation program. After having entered the proliferating basal keratinocytes, HPV gene expression is low and vegetative replication dramatically increases only in the more differentiated layers of the epithelium that are bound to desquamate shortly. The minor levels of protein expression in the lower epithelial layers, the well-directed non-cytolytic genome amplification restricted to the differentiated layers, and the lack of a viremic phase in the viral life cycle are thought to passively help the virus escape immune recognition [52,53]. Thus, HPV has come to an arrangement with the hostile microenvironment and avoids alerting the immune system.

### 3.2. Suppression of Cell-Autonomous Immunity and Acute Inflammation in Keratinocytes

Although HPVs encode only a limited number of regulatory genes, they engage various active strategies to counteract immune recognition and cell-autonomous immune responses at different levels [54]. Mucosal as well as cutaneous HPV were shown to suppress recognition by the pattern recognition receptor toll-like receptor 9 [55,56]. Mucosal HPV also specifically inhibits interferon (IFN) expression, IFN signaling and downstream responses [57,58,59,60]. The HPV E6 oncoprotein directly targets IFN regulatory factor 3 (IRF3) via direct interaction, while E7 interferes with the anti-viral and pro-apoptotic factor IRF1 [61,62,63,64]. Notably, HPV8 has even adapted to an IFN regulatory factor, IRF7, which is activated by UV-light in skin [65]. IRF7 is expressed in suprabasal keratinocytes and it has been demonstrated that it increases HPV8 late promoter activity [66]. In contrast, IRF3-activators, such as dsRNA or RNA bearing 5′ phosphates, efficiently repress HPV8 promoter activity and HPV8 E6 does not counteract the suppressive activity of IRF3 as expected from mucosal HPV-encoded E6 proteins [66]. The IRF3-induced state of cell-autonomous immunity against cutaneous β-HPV in keratinocytes was shown to prevail over IRF7 activity. Thus, IRF3 remains an Achilles heel of this cutaneous virus [66] opening the possibility to use IRF3-activating compounds for anti-viral immunotherapy against β-HPV infection.

In contrast to RNA- and other dsDNA-viruses like herpes-simplex or vaccinia viruses that activate the inflammasome in keratinocytes [67], mucosal HPVs rather dampen acute inflammatory responses in the epithelium. Keratinocytes harboring episomal HPV and cervical cancer cells harboring integrated HPV genomes display only low cytokine and chemokine expression in vitro [68,69,70] and in vivo [12,71] as a direct consequence of HPV oncoprotein-mediated suppression [71,72,73,74]. Mechanistically, it was shown that mucosal HPV oncoproteins target the p300/CBP-associated factor/nuclear factor (NF)-κB pathway [70,74,75,76] and abrogate post-translational processing and secretion of the key inflammatory cytokine interleukin (IL)-1β [77].

In contrast to IL-1β, IL-1α, an intracellularly stored alarmin, is detectable throughout cervical carcinogenesis [78]. IL-1α appears not to be affected by mucosal HPV and this can be exploited for novel immunotherapy strategies. Stimulation of cervical cancer cells with the dsRNA analog PolyIC mimicking dsRNA-virus infection leads to efficient IL-1α release via induction of necroptosis and this was shown to potentiate dendritic cell activation [79]. Notably, PolyIC-induced necroptosis, IL-1α release, and dendritic cell activation are completely dependent on the expression of receptor-interacting protein kinase 3 (RIPK3) in the HPV-transformed cells. In different cervical cancer patients, RIPK3 is expressed at individual levels in the neoplastic cells in situ, which may critically influence their response to dsRNA treatment. Thus, pre-therapeutic RIPK3 expression levels could be used as a novel biomarker to predict the response to immunotherapy with dsRNA or dsRNA-analogs [79,80].

Notably, oncolytic viruses are currently under investigation for cancer therapy and it has become clear that immune activation is an important part of their anti-tumor activity [81]. Whether the immunostimulatory activity of dsRNA oncolytic viruses requires RIPK3 expression in the target cells similar to PolyIC is currently unknown and will be interesting to study.

### 3.3. Suppression of the Recruitment of Professional APC

Professional antigen-presenting cells connect innate and adaptive immunity. APCs migrate to secondary lymphatic tissues where they encounter specific T cells, which subsequently become activated and are redirected to the sites of infection. Local factors released from epithelial cells critically influence recruitment, differentiation and activation of APCs. Although the role of murine Langerhans cells has been controversially discussed, human Langerhans cells have been shown to prime and cross-prime naive CD8^+^ T cells [82], which are known to be critical for the immune control of HPV infection.

Evidence is emerging that HPV infection actively interferes with human Langerhans cell homeostasis in the epidermal compartment. Both cutaneous and mucosal HPV-infected epithelia harbor only low numbers of Langerhans cells [71,83] and the responsiveness of in vitro-generated human Langerhans cells to virus-like particles appears to be restricted [84]. Intriguingly, the expression of chemokines attracting APCs to the epithelium, such as C-C chemokine ligand (CCL)20 and CCL2, were found to be particularly low in HPV-infected epithelia in vivo and in vitro-studies demonstrated that this results from HPV oncoprotein-mediated suppression [11,12,71,72,73,74]. While mucosal HPV oncoproteins target the Langerhans attracting chemokine CCL20 by interfering with the NF-κB pathway [74], cutaneous β-HPV employ a different strategy to suppress CCL20 [71]. In skin, stress signals like UV-light can lead to the depletion of Langerhans cells from the epidermis [85]. Subsequent epithelial up-regulation of the chemokine CCL20 leads to C-C chemokine receptor 6 (CCR6)-dependent repopulation of the skin with CD1a^+^ Langerhans cell precursors [86]. In normal human keratinocytes, the differentiation-associated transcription factor C/EBPβ has been identified as a novel regulator of CCL20 expression and in human skin. Both C/EBPβ and CCL20 are expressed in the uppermost nucleated epithelial layers. In HPV8-infected skin, however, CCL20 is almost lacking. It has been shown that the HPV8 E7 oncoprotein directly interacts with C/EBPβ in keratinocytes and interferes with its binding to chromatin within the CCL20 promoter region. This suppresses CCL20 expression. As a consequence, Langerhans cell migration is inhibited preventing repopulation of the epithelium with these important APCs [71].

Thus, HPV infection actively suppresses cell-autonomous viral recognition and acute inflammatory signaling in the host keratinocyte as well as recruitment of epithelial APCs. The low levels of inflammatory cytokines produced by HPV-infected cells may further contribute to the lack of APC activation, eventually allowing the virus to escape from local immunosurveillance and to persist in the epithelium.

## 4. Immunopathogenesis of Transforming HPV Infection during Progression to Invasive Cancer

### 4.1. Chronic Stromal Inflammation during Progression to Cancer

There is ample evidence that oncogenic HPV infection in the human cervix and skin starts with expansion of the epithelial stem cell compartment [25,87], which may provide a particularly vulnerable and immune privileged milieu [88,89,90]. Moreover, HPV blocks acute NF-κB- and C/EBPβ-dependent inflammatory signaling in host keratinocytes as outlined above. In persistent low-grade lesions inflammatory cells are barely detectable.

However, with increasing dysplasia a dramatic increment of stromal infiltration with immune cells is noted in cervical patient biopsies [11,12,13,83,91]. From other cancers it has become clear that chronic inflammation can fuel immune deviation [14,92,93,94] and the selection pressure set by the local microenvironment can greatly impact the outcome of the neoplastic process [95]. Since HPV oncoproteins suppress acute inflammatory responses [71,72,73,74,76] and HPV-positive cancer cells produce only low chemokine levels [68,69,96], the mechanisms underlying immune cell recruitment remained unclear for a long time.

### 4.2. Paracrine IL-6 Instructs Myelomonocytic Cells to Create a Pro-Tumorigenic and Immunosuppressive Microenvironment in Cervical Carcinogenesis

A clue came from the observation that HPV-transformed cells potently up-regulate chemoattractants in the tumor stroma [11,12]. In monocytes they induce CCL2 production in the nanogram range [11]. This is supposed to attract further myelomonocytic cells and to sustain the inflammatory microenvironment via a CCR2-dependent autocrine amplification loop. Strikingly, CCL2 also leads to a tremendous production of the matrix-metalloproteinase (MMP)-9 in monocytes via intracellular Ca^2+^-signaling. MMP-9 has been detected in monocytes starting to infiltrate cervical high-grade lesions at the switch to malignancy [11]. Local production of MMP-9 is a particularly interesting consequence of CCL2/CCR2 stimulation. It can promote vasculogenesis and trigger the angiogenic switch during carcinogenesis required for tumor growth [97]. Transgenic mouse models have provided evidence that myeloid cell-derived MMP-9 expression promotes HPV-driven carcinogenesis, and blockage of MMP-9 strongly impairs HPV oncogene-driven carcinogenesis in mice [98,99]. Importantly, in cervical cancer patients high MMP expression correlates with a poor prognosis [100].

Neutralization experiments revealed that HPV-transformed cells induce CCL2 and subsequent MMP-9 expression in monocytes via a combination of IL-6 and macrophage colony-stimulating factor (M-CSF). They activate the janus kinase/signal transducer and activator of transcription 3 (JAK/STAT3) signaling pathway in monocytes and various JAK/STAT3-inhibitors are able to interfere with this pro-tumorigenic response [11].

During productive infection HPV suppresses IL-6 similar to other cytokines [70]. However, both “switch factors” IL-6 and M-CSF, that are necessary for the pro-tumorigenic response in monocytes, are highly up-regulated during later stages of human cervical carcinogenesis in situ [69,101]. Clinically most relevant, IL-6 expression is associated with a negative prognosis for cervical cancer patients [102] further highlighting its pivotal role in linking chronic inflammation and progression to invasive cancer [103].

Stromal myelomonocytic cells can either differentiate into dendritic cells, APCs destined to mount adaptive immune responses, or into macrophages, tissue-resident phagocytes. For the initiation of adaptive immunity, dendritic cells have to mature as indicated by surface expression of CD83, to up-regulate major histocompatibility complex class I and II required for antigen presentation as well as co-stimulatory molecules such as CD80 and CD86 required for T-cell activation, and finally to produce cytokines that polarize T helper 1 (Th1) cells required for efficient CD8^+^ cytotoxic T cell responses. Under normal conditions, the migration receptor CCR7 becomes expressed on their surface during maturation, ensuring their responsiveness to lymph node homing chemokines [104,105]. A second migration factor, MMP-9, is needed to allow migration through the extracellular matrix [106,107]. In cervical cancer patients, mature CD83^+^ dendritic cells are present in the tumor stroma. However, they were found to be largely devoid of CCR7 expression [13]. As the underlying mechanism, it was shown that cervical cancer cells actively interfere with NF-κB activation in CD83^+^ phenotypically mature dendritic cells. As a consequence, expression of the chemokine receptor CCR7 is suppressed in the dendritic cells and their migration towards lymph node homing chemokine is blocked [13]. This may lead to an impaired antigen transport to secondary lymphoid tissues by stromal dendritic cells in cervical cancer patients. Moreover, in high-grade lesions and invasive cancers only low IL-12p40 expression levels required to mount Th1 responses and a shift from Th1 to Th2 responses are observed [108,109].

In contrast to CCR7, MMP-9 is potently up-regulated in immature and mature dendritic cells. Notably, both CCR7 and MMP-9 up-regulation are mediated by IL-6 from the cervical cancer cells [13,110]. Thus, cervical cancer-derived IL-6 immobilizes dendritic cells in the tumor stroma via CCR7 suppression facilitating local MMP-9 production.

In cervical cancer stroma M2-polarized macrophages accumulate with low IFN-γ production and a low capacity to stimulate T cell proliferation [111]. M2 macrophages are also supposed to have a negative impact on cervical cancer therapy, such as therapy with immunoglobulin G (IgG) antibodies directed against epidermal growth factor receptor (EGFR) [112]. Notably, anti EGFR-specific IgA antibodies may overcome this obstacle, since they can engage tumor-associated myeloid cells for tumor cell killing [113]. This may provide a clear advantage for treatment of M2-infiltrated tumors and therefore IgA antibodies might represent a novel future category of antibodies for targeted tumor therapy.

Besides neoplastic cells, M2-polarized macrophages were also found to express the programmed death-ligand 1 (PD-L1) [114]. There is clear evidence that cytotoxic T-lymphocyte-associated protein (CTLA)-4/CD28 and PD-1/PD-L1 interactions between T cells and other cells exert suppressive signals (immune checkpoints) limiting T cell function and maintaining self-tolerance. This has led to regulatory approval and successful implementation of blocking antibodies targeting CTLA-4, PD-1, and PD-L1 for the treatment of different malignancies. PD-1 was detected on most infiltrating CD8^+^ T cells in cervical cancer [115] suggesting that M2 macrophages might contribute to suppression of cytotoxic T cell responses. Preliminary studies with the PD-1 blocker pembrolizumab, however, showed only low response rates in cervical cancer patients with advanced disease. Currently, predictive biomarkers allowing more precise patient selection are still lacking and it is expected that efficacy might increase when these blockers are applied in combination with other immunotherapies, such as therapeutic vaccines [16,116]. Notably, it has been shown that M2 macrophage differentiation is driven by cervical cancer-derived IL-6 together with prostaglandin E2 [111].

From these studies evidence is increasing that paracrine IL-6 as well as subsequent STAT3 activation and NF-κB suppression are central for reprogramming myelomonocytic cells creating a pro-tumorigenic and immunosuppressive microenvironment in cervical carcinogenesis. As a consequence, phenotypically mature but functionally impaired dendritic cells and macrophages are actively retained in the tumor stroma and this may (1) prevent the initiation of anti-tumoral adaptive Th1 immune responses; (2) suppress cytotoxic T cell activity; and (3) contribute to an aberrant local expression of MMP-9 that promotes tumor growth and vasculogenesis. This suggests that the IL-6/JAK/STAT3 signaling pathway might be an interesting target to revert immune deviation in cervical cancer. In fact, the IL-6/JAK/STAT3 signaling pathway is “druggable” at various levels [117] and clinical trials for different cancer entities are ongoing (see clinicaltrials.gov).

### 4.3. Paracrine IL-6 Induces CCL20 Chemokine Expression in Stromal Mesenchymal Cells to Support Th17 Recruitment

In human biopsies of cervical carcinogenesis, enhanced infiltration of Th17 cells is observed with increasing stages of disease and this correlates with up-regulation of CCL20 expression in the stromal mesenchymal compartment [12]. It is well known that Th17 recruitment is mediated by CCL20 in a CCR6-dependent manner [118]. Th17 infiltration starts in precursor lesions, and in invasive cancers a high Th17/Treg ratio is observed [119,120]. Th17 cells are a particular subset of the CD4^+^ T cell lineage that can exert either regulatory or inflammatory functions [121]. In various different cancers Th17 cells promote tumor growth, angiogenesis and also the recruitment of further inflammatory immune cells [122,123,124]. Mechanisms underlying stromal CCL20 expression remained unclear until cervical cancer explant cultures revealed that cancer-associated fibroblasts produce enormous amounts of CCL20 [12]. These fibroblasts display an activated phenotype characterized by enhanced C/EBPβ expression. C/EBPβ is also known as the NF-IL6 transcription factor and is inducible by pro-inflammatory cytokines like IL-6 [125]. Recently, C/EBPβ has been identified as a novel transcriptional regulator of CCL20 [71]. Thorough analysis demonstrated that cervical cancer-derived IL-6 drives C/EBPβ-mediated CCL20 induction in cancer-associated fibroblasts and CCL20/CCR6-dependent Th17 recruitment [12]. This further substantiates the key role of paracrine IL-6 in cervical carcinogenesis for the initiation and maintenance of chronic inflammation and tumor progression.

These studies provided evidence that paracrine IL-6 shapes a proinflammatory and immunosuppressive microenvironment in cervical carcinogenesis via activation of two pathways in stromal immune and stromal mesenchymal cells, the JAK/STAT3 and the C/EBPβ signaling pathway. In HPV-driven carcinogenesis, IL-6-induced JAK/STAT3- and C/EBPβ-driven stromal inflammation may play a key role in promoting tumor progression. At the same time paracrine IL-6 can limit NF-κB-dependent anti-viral and anti-tumor immune responses in APCs, further highlighting IL-6 as an attractive target for adjuvant immunotherapy of cervical cancer.

### 4.4. Regulation and Consequences of Epithelial JAK/STAT3 Pathway Activation in HPV-Induced Carcinogenesis

In HPV-driven carcinogenesis STAT3 is not only activated in the tumor microenvironment. Also, epithelial cells in cervical high-grade lesions display strong pTyr705-STAT3 activation by far exceeding activation levels in normal exocervical epithelium or low-grade lesions [11,126]. Studies with transgenic mice expressing the HPV8 early region under the K14-promoter have provided evidence that epithelial STAT3 activation is necessary for HPV8-driven skin tumorigenesis [127] and this may also apply to human HPV-associated carcinogenesis at other body sites.

Notably, in invasive cervical cancers STAT3 activation is often retained at the tumor margin adjacent to the stroma, while in other parts of the tumor STAT3 activation declines compared to cervical high-grade lesions [126]. This suggests a paracrine mode of STAT3 activation at the tumor invasive margin. The overall decline of STAT3 activity in cervical cancer cells can be explained by a decline of the IL-6 binding receptor chain gp80 in cervical cancer cells [69]. Correspondingly, non-malignant HPV-transformed cells strongly respond to IL-6 alone. In cultured cervical cancer cells, however, efficient STAT3 activation can only be elicited by IL-6 in the presence of soluble gp80 (sgp80) [69,126,128].

Strikingly, when the IL-6/STAT3-signaling pathway is activated in cervical cancer cells, they are more efficiently killed by chemotherapeutic drugs, such as cisplatin or etoposide [126]. This was unexpected, since in various other human malignancies STAT3 activation promotes tumor growth and resistance to chemotherapy [129]. As the underlying mechanism, dramatic up-regulation of the pro-apoptotic factor IRF1 has been identified [126]. Obviously, IL-6/STAT3-induced IRF1 activity prevails over the HPV oncogene-mediated inhibition of IRF1 [61,62,63]. Of most clinical relevance, epithelial IRF1 expression in pre-therapeutic biopsies of cervical cancers significantly correlates with the patient`s response to chemo- or radiochemotherapy [126]. This strongly indicates that pre-therapeutic IRF1 expression could be of high value as a novel biomarker to predict response to chemo- or radiochemotherapy in patients [126].

In summary, targeting the IL-6/JAK/STAT3-pathway emerges as a promising way to improve the immune microenvironment in cervical carcinogenesis. However, recent data suggest that these compounds can also interfere with STAT3-induced IRF1 expression in HPV-positive cancer cells, thereby increasing their resistance to chemo- or radiochemotherapy. Therefore, the timing of IL-6/JAK/STAT3-inhibitor administration during therapy appears to be of critical importance. They should not be applied prior to but rather after chemo- or radiochemotherapy of HPV-driven cancers.

## 5. Conclusions and Prospects for Diagnosis and Immunotherapy

Over recent years it has become evident that the local microenvironment and particularly the immune system plays a pivotal role in carcinogenesis [93,94]. In various cancer types it has been shown that immune cells have a significant prognostic impact, and in colon cancer an “immunoscore” quantifying in situ immune cell infiltrates seems to be superior to the TNM classification [130,131]. Moreover, immune checkpoint inhibitors interfering with PD-1 or CTLA-4 pathways were recently shown to improve therapy response rates in various cancers including melanoma, non-small cell lung cancer, head and neck cancer, renal cell carcinoma and Hodgkin’s lymphoma, and numerous clinical trials are ongoing [132]. This has also changed the virologist’s view on cancer progression from the strict focus on viral oncogenes necessary for carcinogenesis to a more complex view of the interaction between tumor viruses, the immune system and the tumor microenvironment in promoting cancer progression.

In HPV-induced carcinogenesis, there is now ample evidence for a stage-specific interplay between virally-infected keratinocytes and the local immune microenvironment that can determine the course of disease (Figure 1). This knowledge will pave the way for (1) novel diagnostic tools including immunoscores that allow the discrimination of non-progressing and progressing precursor lesions; (2) novel biomarkers that improve the prediction of therapy response, such as IRF1, which indicates response to chemo- or radiochemotherapy; and (3) novel immunotherapeutic approaches beyond checkpoint inhibitors that target critical stage-specific mechanisms and pathways in HPV-driven carcinogenesis. These include IRF3-activating compounds such as dsRNA to combat oncogenic β-HPV infection, dsRNA-based immunotherapies for patients with high intratumoral RIPK3 levels, IgA-based antibody therapies that engage myeloid cells for tumor cell killing, and last but not least IL-6/JAK/STAT3-pathway blocking regimens for cervical cancer patients after treatment with chemo- or radiochemotherapy.
